# Supplementary material for: Sea ice–air interactions amplify multidecadal variability in the North Atlantic and Arctic region
Source: Nat Commun. 2022 Apr 19;13:2100. doi: 10.1038/s41467-022-29810-7 (PMC9018715; doi:10.1038/s41467-022-29810-7)
Supplement: Supplementary file 1 — Supplementary Information [file 41467_2022_29810_MOESM1_ESM.pdf]

Supplementary information for

Sea ice-air interactions amplify multidecadal variability in the North Atlantic and Arctic region

Jiechun Deng and Aiguo Dai

Supplementary information includes:

- Supplementary Table 1
- Supplementary Figures 1–19, whose acronyms are defined in Figs. 1 and 5.

Supplementary Table 1. Data availability of CMIP5 (in normal font) and CMIP6 (in *italic* font) model simulations used in this study. Regular atmospheric and ocean variables (e.g., Tas, SST, and SIC) are available during 1900–2300 for all of the twelve models, except for meridional overturning circulation (MOC; including *msftmyz*, *msftmz*, or *msftyz* in the standard CMIP5 and CMIP6 notations) and mixed layer depth (MLD; *mlotst* in the CMIP5 and CMIP6 notations) (see the rightmost two columns below). Note: √ = available, "–" = unavailable.

No.	Model name	Extended RCP8.5 to 2300	MOC data	MLD data
1	bcc-csm1-1	√	–	–
2	CCSM4	√	√	–
3	CNRM-CM5	√	√	√
4	GISS-E2-H	√	–	–
5	GISS-E2-R	√	√	–
6	HadGEM2-ES	√	–	–
7	IPSL-CM5A-LR	√	–	–
8	MPI-ESM-LR	√	√	√
9	*CanESM5*	√	–	–
10	*CESM2-WACCM*	√	√	√
11	*IPSL-CM6A-LR*	√	√	√
12	*MRI-ESM2-0*	√	√	√

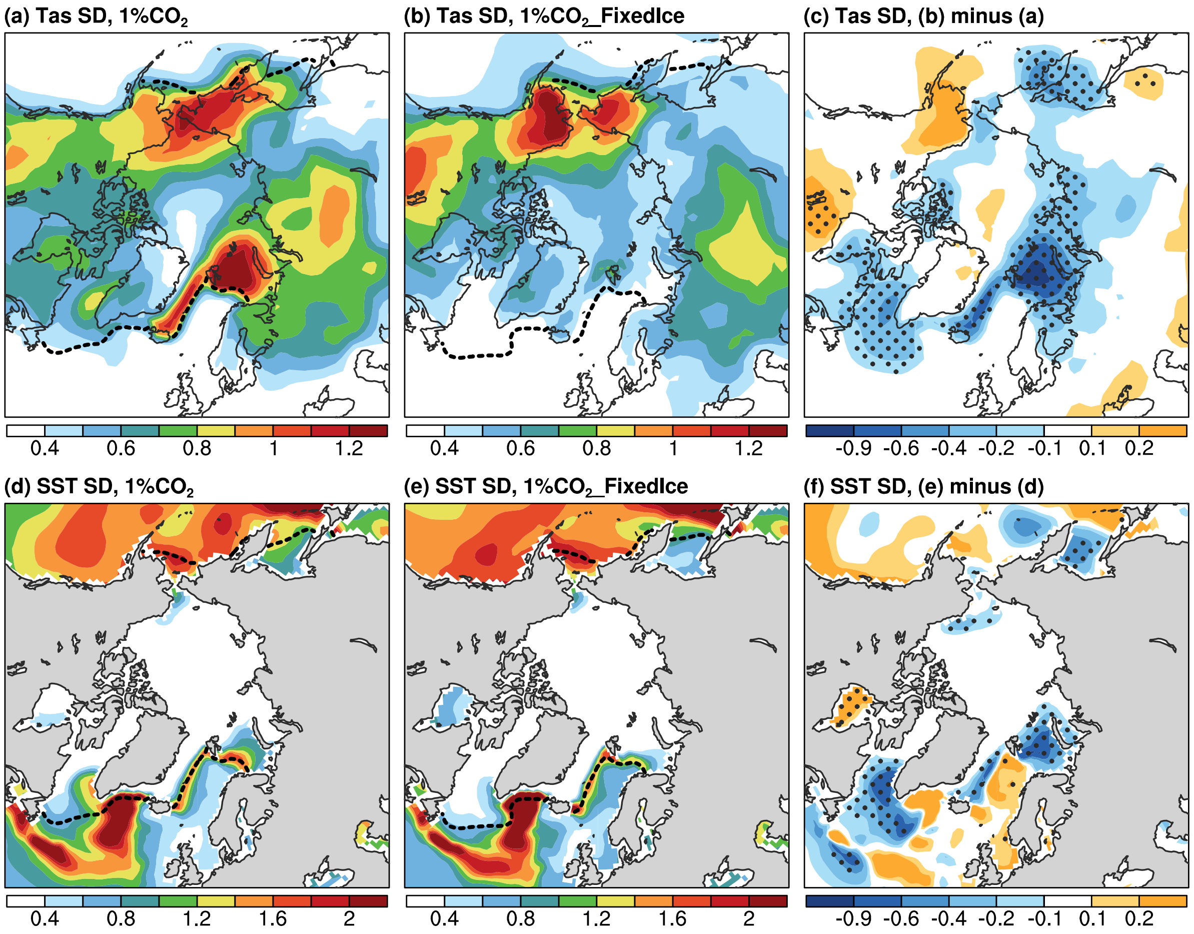

Supplementary Figure 1. CESM1-simulated multidecadal temperature variability and its sea ice-induced difference under increasing CO_2. (**a**–**c**) Distributions of the standard deviation (SD) of the 10–90-year band-pass filtered DJF-mean surface air temperature (Tas) anomalies (in °C) north of 50°N from the CESM1 (**a**) 1%CO_2 and (**b**) 1%CO_2_FixedIce runs and (**c**) their difference (i.e., 1%CO_2_FixedIce minus 1%CO_2) during years 11 to 150 with the forced signal removed (see Methods). (**d**–**f**) Same as (**a**–**c**) but for the similarly-filtered DJF-mean sea surface temperature (SST) anomalies (in °C; multiplied by 5 as in Fig. 2). The black dashed contour in **a** and **d** (**b** and **e**) denotes the climatological DJF-mean sea-ice edge (for sea-ice concentration =10%) over the same period from 1%CO_2 (1%CO_2_FixedIce). The stippling in **c** and **f** indicates that the SD difference is statistically significant at the 5% level based on a *F*-test.

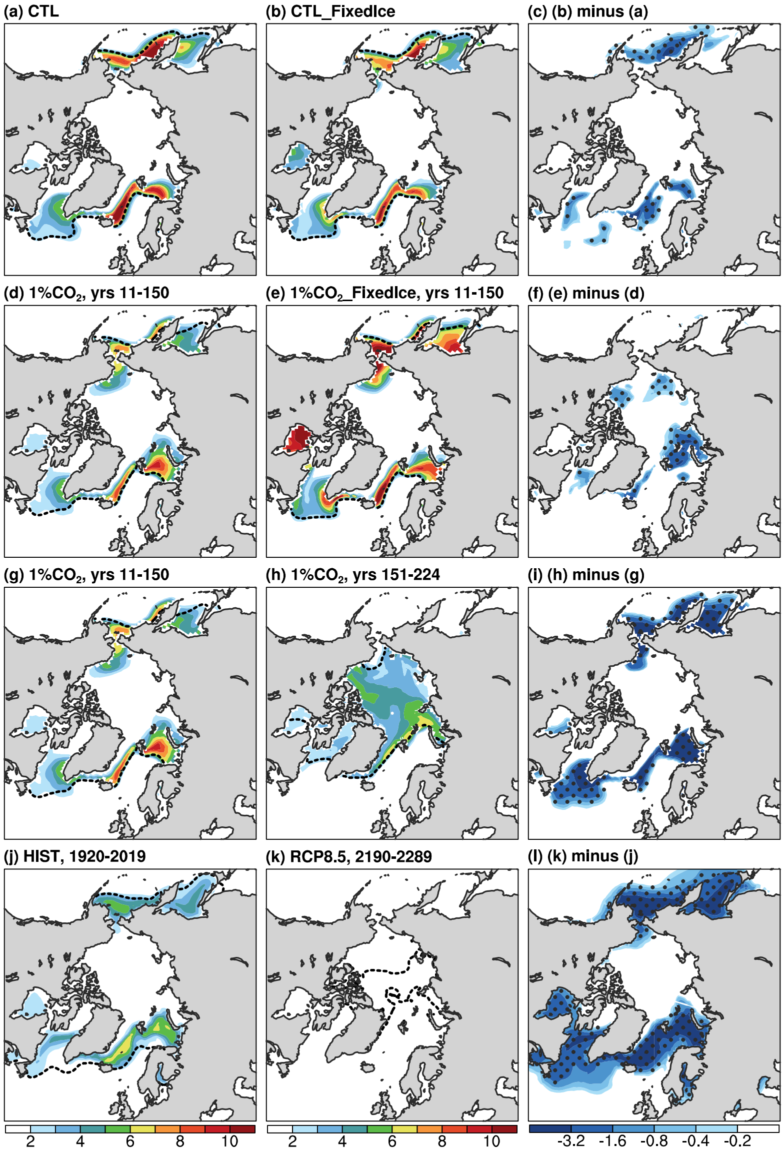

Supplementary Figure 2. Differences in multidecadal sea-ice cover (SIC) variability in CESM1 and CMIP5/6 simulations. (**a**–**c**) Distributions of the standard deviation (SD) of the 10–90-year band-pass filtered DJF-mean SIC anomalies (in % of area) north of 50°N from the CESM1 (**a**) CTL and (**b**) CTL_FixedIce runs and (**c**) their difference (**b** minus **a**) from years 11–490. (**d**–**f**) Same as (**a**–**c**), but for the SIC SD from the CESM1 (**d**) 1%CO_2 and (**e**) 1%CO_2_FixedIce runs and (**f**) their difference (**e** minus **d**) during years 11–150 with the forced signal removed. (**g**–**i**) Same as (**a**–**c**), but for the SIC SD from the CESM1 1%CO_2 run during (**g**) years 11–150 and (**h**) years 151–224 with the forced signal removed and (**i**) their difference (**h** minus **g**). (**j**–**l**) Same as (**g**–**i**), but for the SIC SD averaged over twelve CMIP5 and CMIP6 models during (**j**) 1920–2019 and (**k**) 2190–2289 with the forced signal removed and (**l**) their difference (**k** minus **j**). The black dashed contour in the left and middle columns denotes the corresponding climatological DJF-mean sea-ice edge (for sea-ice concentration=10%) over the respective time period. The stippling in the right column indicates that the SD difference is statistically significant at the 5% level based on a *F*-test in panels (**c**,**f**,**i**) and that at least 8 out of 12 models agree on the sign of change in panel (**l**).

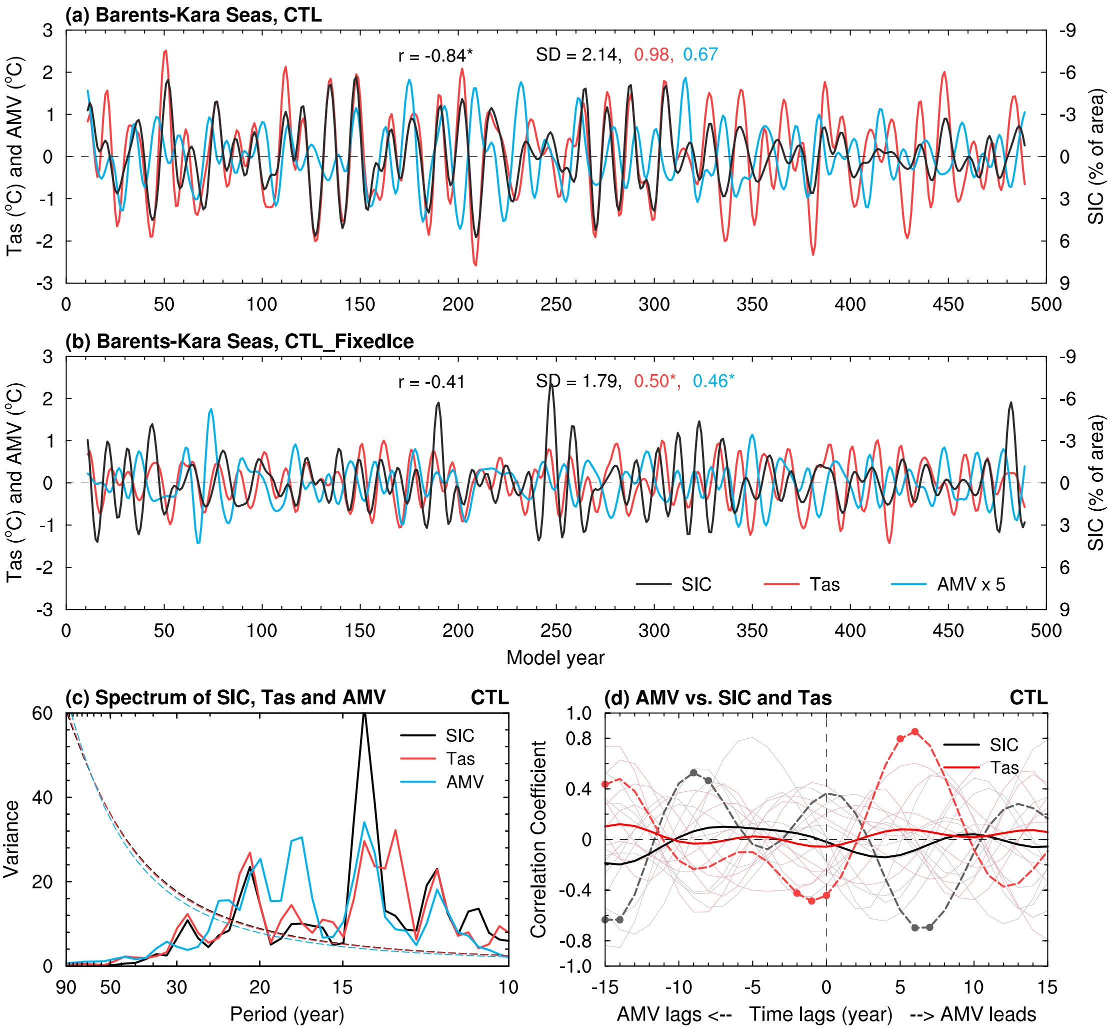

Supplementary Figure 3. Relationships among Atlantic Multidecadal Variability (AMV), BKS sea ice cover (SIC) and surface air temperature (Tas) in CESM1. Filtered time series of DJF-mean anomalies of SIC (black; in % of area; right *y*-axis, increases downward) and Tas (red; in °C, left *y*-axis) averaged over the Barents-Kara Seas (BKS) defined in Fig. 1b and the AMV index (blue; in °C, multiplied by 5 in order to use the same left *y*-axis) from the CESM1 (**a**) CTL and (**b**) CTL_FixedIce runs from years 11 to 490. A 10–90-year Lanczos band-pass filter was used. The correlation coefficient (*r*) is between SIC and Tas. The SD of each curve is given in the respective color on (**a-b**). The superscript "*" and "#" indicate the correlation (the SD difference) is statistically significant at the 5% and 10% levels, respectively, based on a resampling technique (a *F*-test) (see Methods). (**c**) Power spectrum (standardized to use the same *y*-axis) of the time series shown in (**a**). The dashed curves are for the 95% confidence bound. (**d**) Lead-lag correlation coefficients of the AMV with SIC (black) and Tas (red) over the BKS from ERA5 reanalysis as shown in Fig. 1d (dashed lines) and the CESM1 CTL run as shown in (**a**) (thick solid lines). The thin solid lines with the respective colors are for nine 50-year (similar to the length of the filtered ERA5 data) segments from CTL, and their ensemble mean are similar to the respective thick solid lines (which are for all years). The dots indicate the correlation coefficient is statistically significant at the 5% level based on a resampling technique (see Methods).

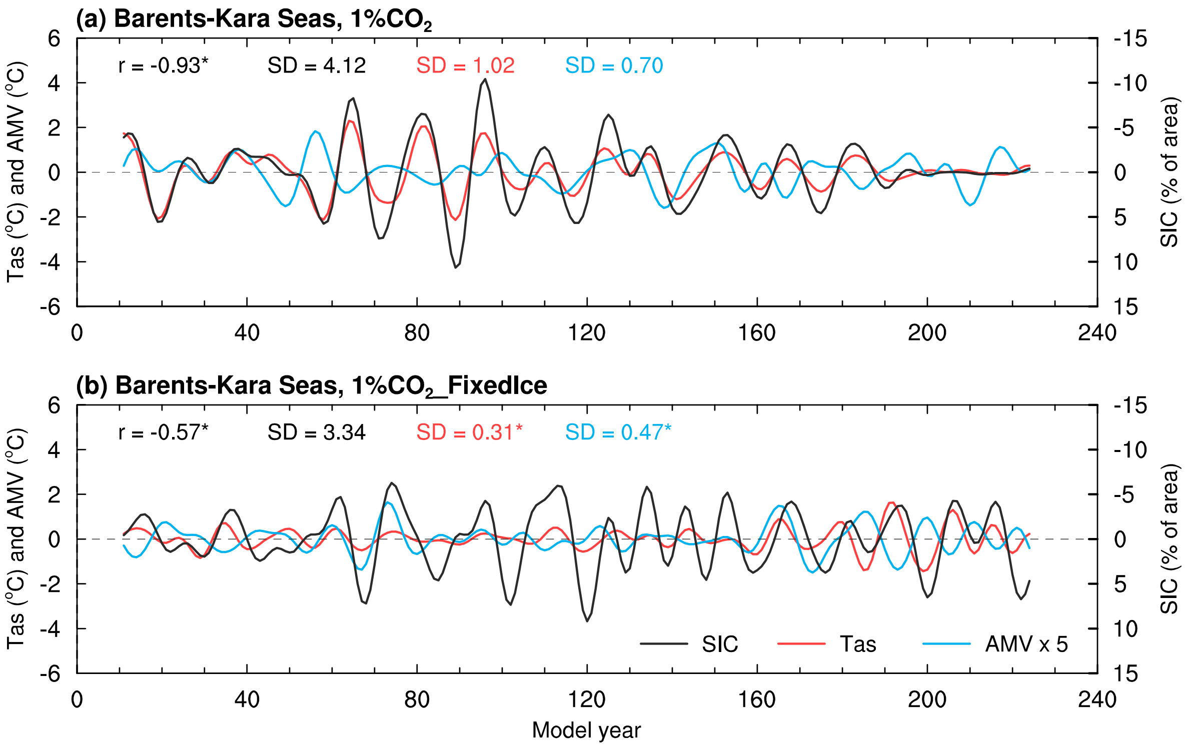

Supplementary Figure 4. CESM1-simulated multidecadal fluctuations in AMV, BKS sea ice and air temperature under increasing CO_2. Filtered time series of DJF-mean anomalies (with the forced signal removed; see Methods) of SIC (black; in % of area; right *y*-axis, increases downward) and Tas (red; in °C, left *y*-axis) averaged over the Barents-Kara Seas (BKS) as defined in Fig. 1b and the AMV index (blue; in °C, multiplied by 5 to use the same left *y*-axis) from the CESM1 (**a**) 1%CO_2 and (**b**) 1%CO_2_FixedIce runs from years 11 to 224. A 10–90-year Lanczos band-pass filter was used. The correlation coefficient (*r*) is between SIC and Tas. The SD of each curve is given in the respective color for years 11–150. The superscript "*" and "#" indicate the correlation (the SD difference) is statistically significant at the 5% and 10% levels, respectively, based on a resampling technique (a *F*-test) (see Methods).

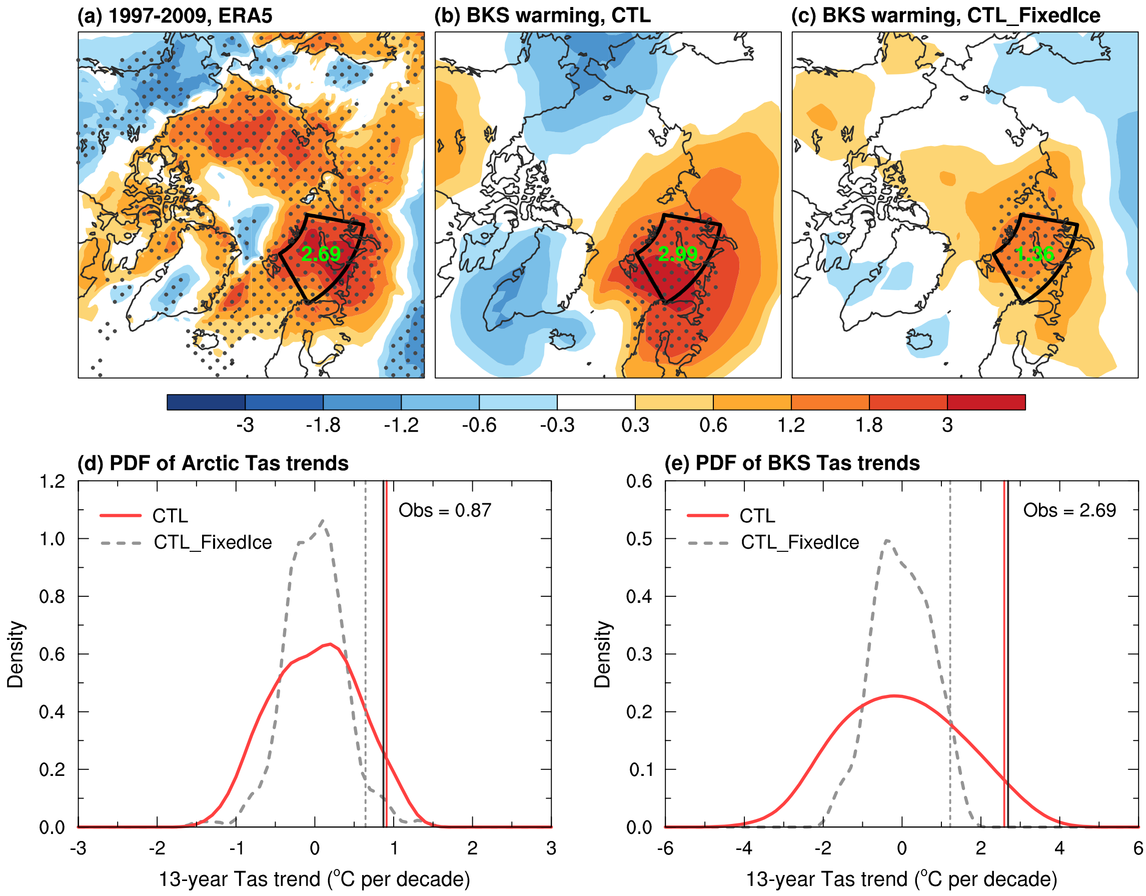

Supplementary Figure 5. Decadal warming trends over the Arctic and BKS from ERA5 and CESM1. (**a**–**c**) Decadal trend maps of 10–90-year band-pass filtered DJF-mean surface air temperature (Tas) anomaly field (in °C per decade) north of 60°N (a) from ERA5 during 1997–2009 (after removing the forced signal, see Methods) and averaged over the 13-year moving periods with the strongest BKS warming trends (top five percentiles) from the CESM1 (**b**) CTL and (**c**) CTL_FixedIce runs during years 11–490. The BKS Tas trend seen in (**a**) ERA5 during 1997–2009 and averaged over the top five percentiles from (**b**) CTL and (**c**) CTL_FixedIce runs are also given within the outlined BKS region (as defined in Fig. 1b). The stippling indicates that the trend is statically significant at the 5% level based on a Students' *t* test in panel **a** or at least 90% of the selected periods agree on the same sign of trend in panels **b**–**c**. (**d**–**e**) The probability density function (PDF) of (**d**) Arctic (north of 65°N) and (**e**) BKS Tas trends over all 13-year moving periods from the CTL (red solid) and CTL_FixedIce (gray dashed) runs based on a gaussian kernel density estimation. The *x* axis is the 13-year Tas trend (in °C per decade) and the *y* axis is the probability density. The vertical black solid lines (with the corresponding value in the top right) indicate the (**d**) Arctic and (**e**) BKS warming trends from ERA5 during 1997–2009, while the vertical red solid and gray dashed lines indicate the 5th percentile values in the CTL and CTL_FixedIce runs, respectively. The occurrence probability for a trend that is similar or larger than the ERA5 trend for the Arctic-mean (BKS) Tas is increased from 2.14% to 5.78% (0.00% to 3.64%) from CTL_FixedIce to CTL. Note that we chose 1997–2009 here because the BKS Tas shows a rapid warming trend over this period (cf. Fig. 1f).

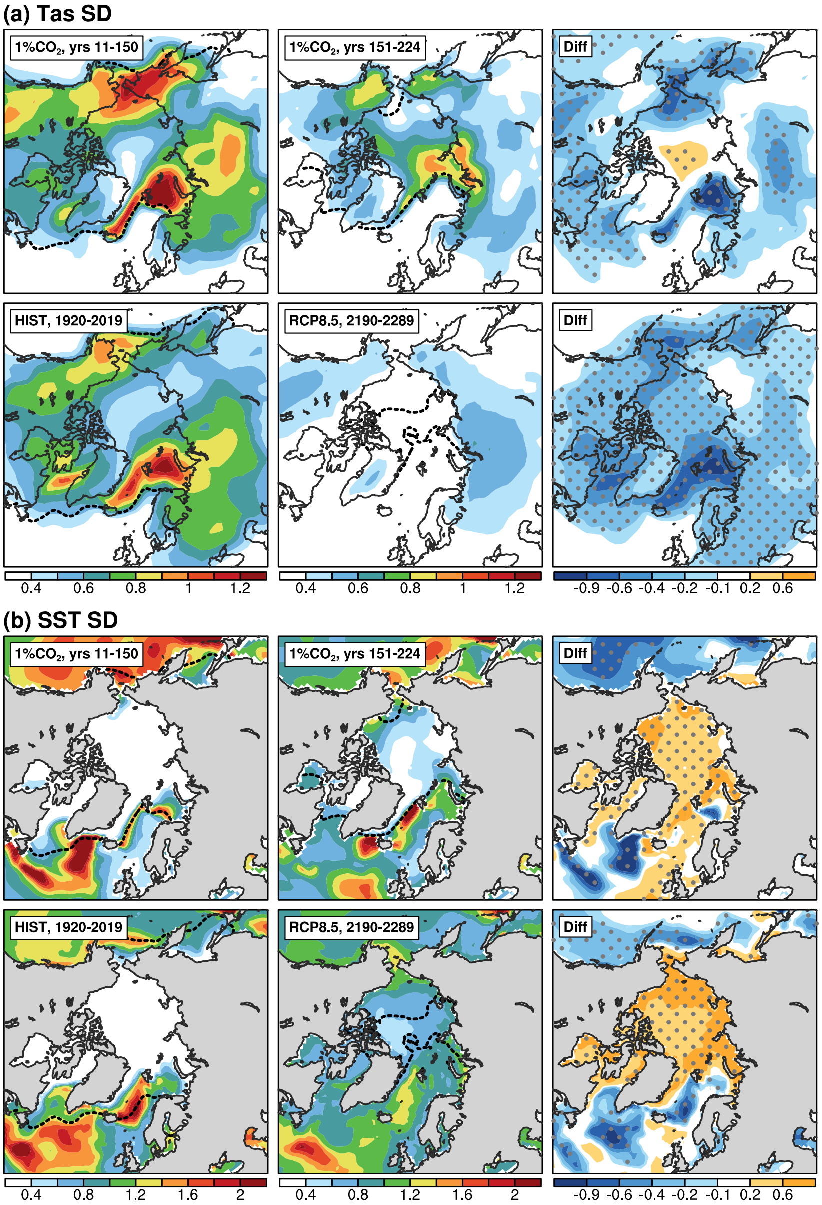

Supplementary Figure 6. Differences in multidecadal temperature variability when sea ice melts away in warm climates. (**a**) Distributions of the SD of the 10–90-year band-pass filtered DJF-mean surface air temperature (Tas) anomalies (in °C) north of 50°N (top row) from the CESM1 1%CO_2 run during (left) years 11–150 and (middle) years 151–224 with the forced signal removed and (right) their difference (i.e., years 151–224 minus years 11–150) and (bottom row) averaged over twelve CMIP5 and CMIP6 models during (left) 1920–2019 and (middle) 2190–2289 with the forced signal removed and (right) their difference (i.e., 2190–2289 minus 1920–2019). The black dashed contour denotes the climatological DJF-mean sea-ice edge (for sea-ice concentration=10%) averaged over the respective period. The stippling indicates that the SD difference is statistically significant at the 5% level based on a F-test for the CESM1 1%CO_2 run or at least 8 out of 12 models agree on the sign of change for CMIP5 and CMIP6 simulations. (**b**) Same as (**a**), but for the similarly-filtered DJF-mean SST anomalies (in °C; multiplied by 5 as in Fig. 2) with the forced signal removed.

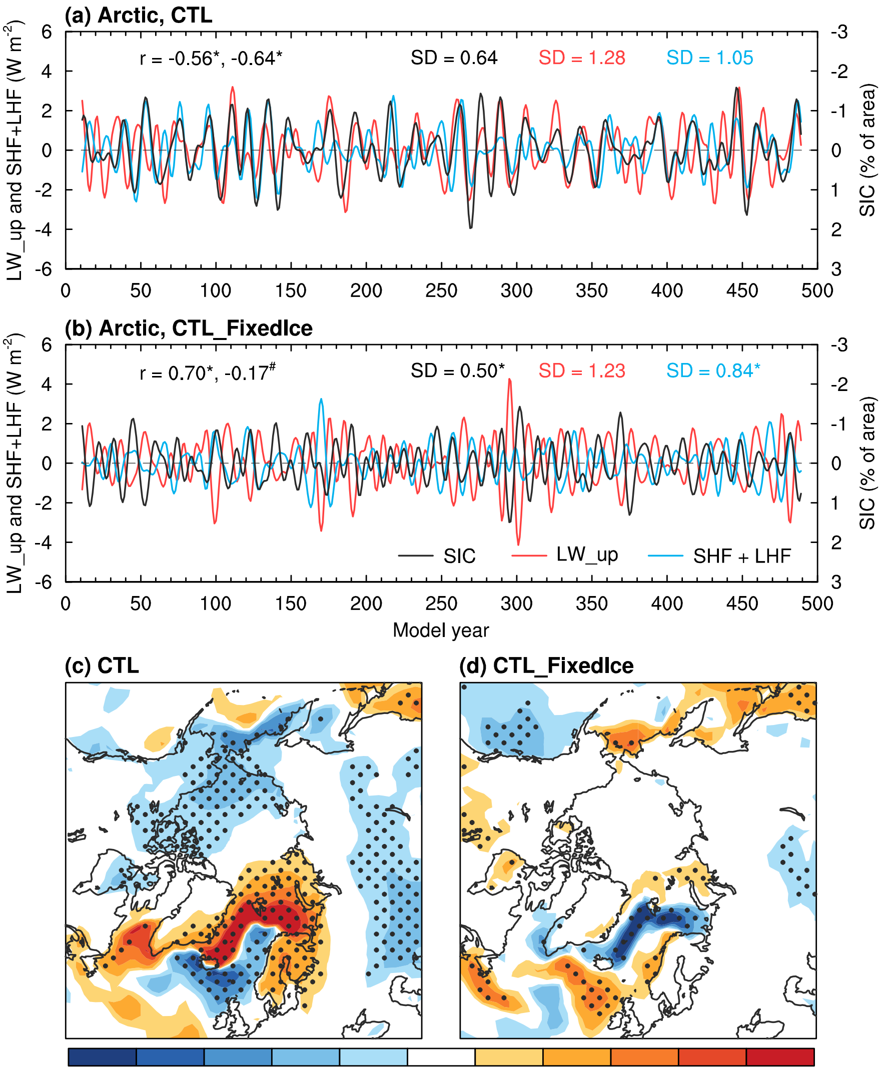

Supplementary Figure 7. Relationships among Arctic sea-ice cover and surface heat fluxes in CESM1. (**a**, **b**) Filtered time series of the DJF-mean anomalies of SIC (black; in % of area; right *y*-axis, increases downward), surface upward longwave radiation (LW_up, red; in W m^{-2}, left *y*-axis), and surface sensible plus latent heat flux (SHF+LHF, blue; in W m^{-2}, left *y*-axis) averaged over the Arctic region north of 65°N from the CESM1 (**a**) CTL and (**b**) CTL_FixedIce runs during years 11–490. A 10–90-year Lanczos band-pass filter was used. The correlation coefficients (*r*) are, from left to right, between SIC and LW_up, and SIC and SHF+LHF. The SD of each curve is also given in the respective color for each panel. The superscript "*" and "#" indicate the correlation (the SD difference) is statistically significant at the 5% and 10% levels, respectively, based on a resampling technique (a *F*-test) (see Methods). (**c**, **d**) Composite differences of the similarly-filtered DJF-mean LW_up + SHF + LHF anomalies (shading, positive upward, in W m^{-2}) north of 50°N between years with low (local minimum smaller than –1 SD) and high (local maximum greater than +1 SD) Arctic SIC anomalies (i.e., low SIC years minus high SIC years) from the CESM1 (**c**) CTL and (**d**) CTL_FixedIce runs from years 11 to 490. The stippling in **c** and **d** indicates the difference is statistically significant at the 5% level based on a Student's *t*-test.

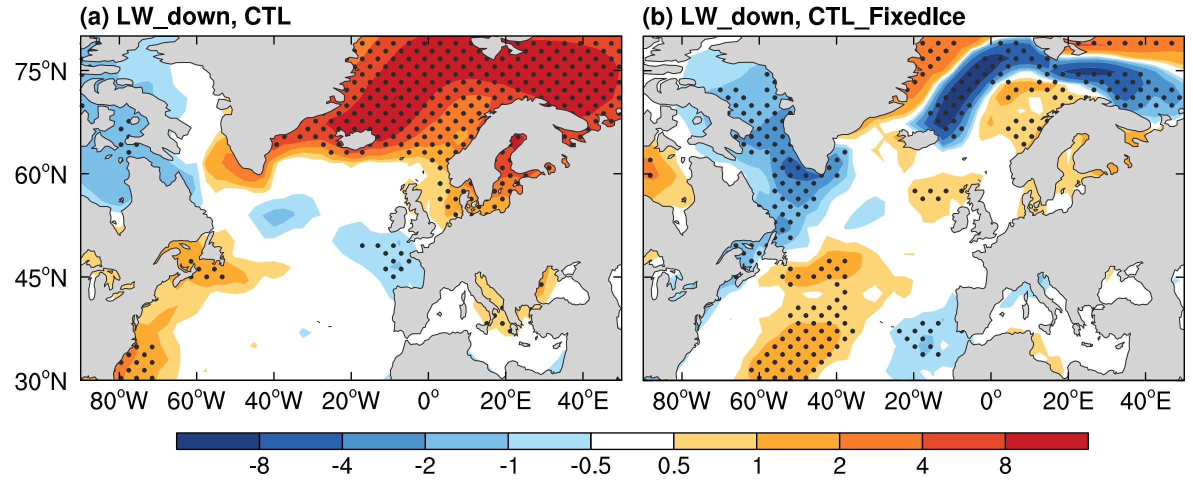

Supplementary Figure 8. Composite differences in downward longwave radiation associated with multidecadal Arctic sea-ice cover (SIC) anomalies in CESM1. Composite differences of the 10–90-year band-pass filtered DJF-mean anomalies of surface downward longwave radiation (LW; shading, in W m^{-2}) over subpolar North Atlantic and Arctic region between years with low (local minimum smaller than –1 SD) and high (local maximum greater than +1 SD) Arctic SIC anomalies north of 65°N (i.e., low SIC years minus high SIC years) from the CESM1 (**a**) CTL and (**b**) CTL_FixedIce runs from years 11 to 490. The stippling indicates the difference is statistically significant at the 5% level based on a Student's *t*-test.

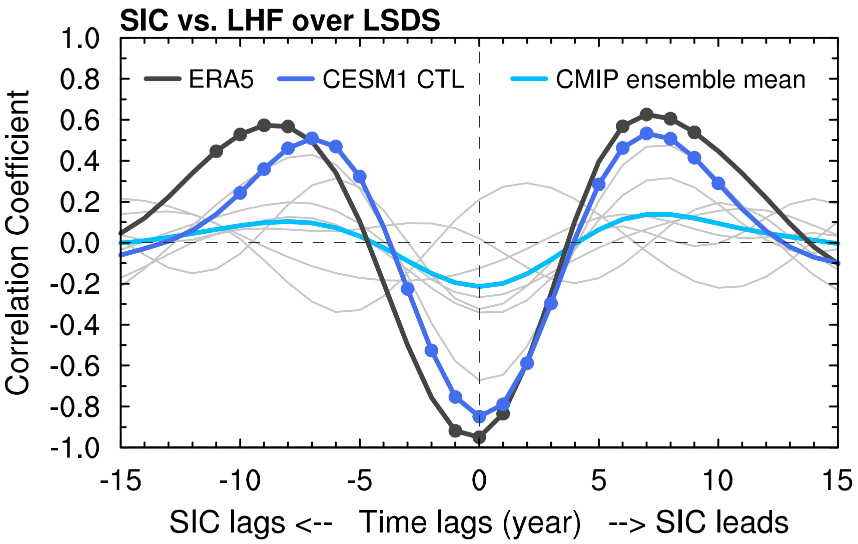

Supplementary Figure 9. Lead-lag correlation between regional sea-ice cover (SIC) and latent heat flux (LHF) from ERA5, CESM1 and CMIP5/6 simulations. Lead-lag correlation coefficients of the DJF-mean band (10–90 year) filtered SIC with LHF averaged over the Labrador Sea and Davis Strait (LSDS, defined in Fig. 1b) from the ERA5 reanalysis during 1950–2019 (black), the CESM1 CTL run (dark blue), and averaged over seven 500-year piControl simulations from seven CMIP5 and CMIP6 models (with the thin gray lines for individual model runs and the light blue line for the ensemble mean). The dots indicate the correlation coefficient is statistically significant at the 5% level based on a resampling technique (see Methods). Note that the LSDS region used here is shifted northward by 10° latitudes (i.e., 45°–65°W, 65°–80°N) in ERA5 with respect to that defined in CTL and CMIP5/6 piControl runs (i.e., 45°–65°W, 55°–70°N) to better show the current SIC-LHF relationship, because the sea-ice edge (and thus SIC variations) over LSDS has retreated further northward under the current climate (cf. Fig. 1b) than that under the pre-industrial climate (cf. Supplementary Fig. 2a).

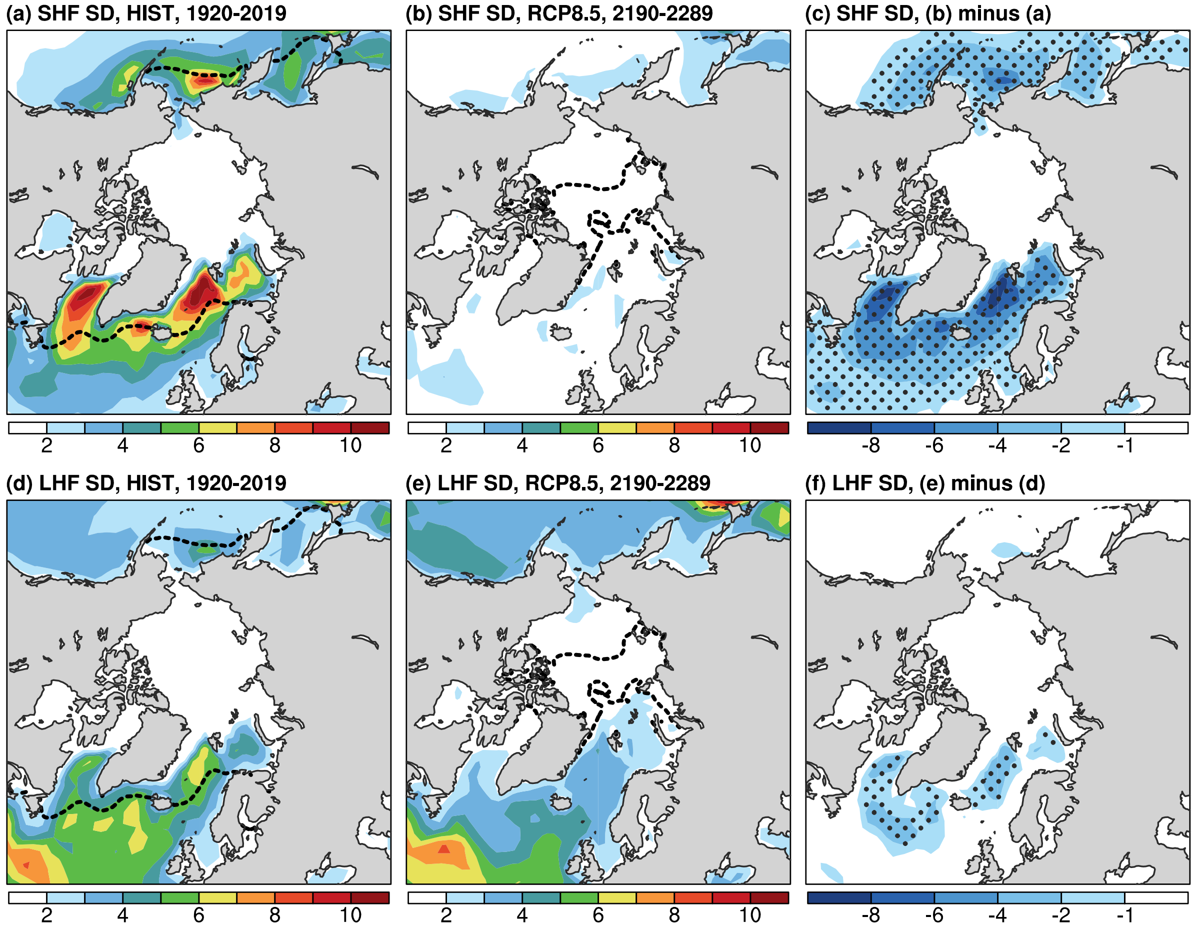

Supplementary Figure 10. Differences in multidecadal variability of surface heat fluxes when sea ice melts away in warm climates. (**a**–**c**) Distributions of the standard deviation (SD) of the 10–90-year band-pass filtered DJF-mean surface sensible heat flux (SHF) anomalies (in W m^{-2}) north of 50°N averaged over twelve CMIP5 and CMIP6 models during (**a**) 1920–2019 and (**b**) 2190–2289 with the forced signal removed (see Methods) and (**c**) their difference (i.e., 2190–2289 minus 1920–2019). (**d**–**f**) Same as (**a**–**c**) but for the similarly-filtered DJF-mean surface latent heat flux (LHF) anomalies (in W m^{-2}) with the forced signal removed. The black dashed contour in **a** and **d** (**b** and **e**) denotes the climatological DJF-mean sea-ice edge (for sea-ice concentration=10%) averaged over twelve models during 1920–2019 (2190–2289). Only negative values are plotted in **c** and **f** for clarity and the stippling indicates that at least 8 out of 12 models agree on the sign of change.

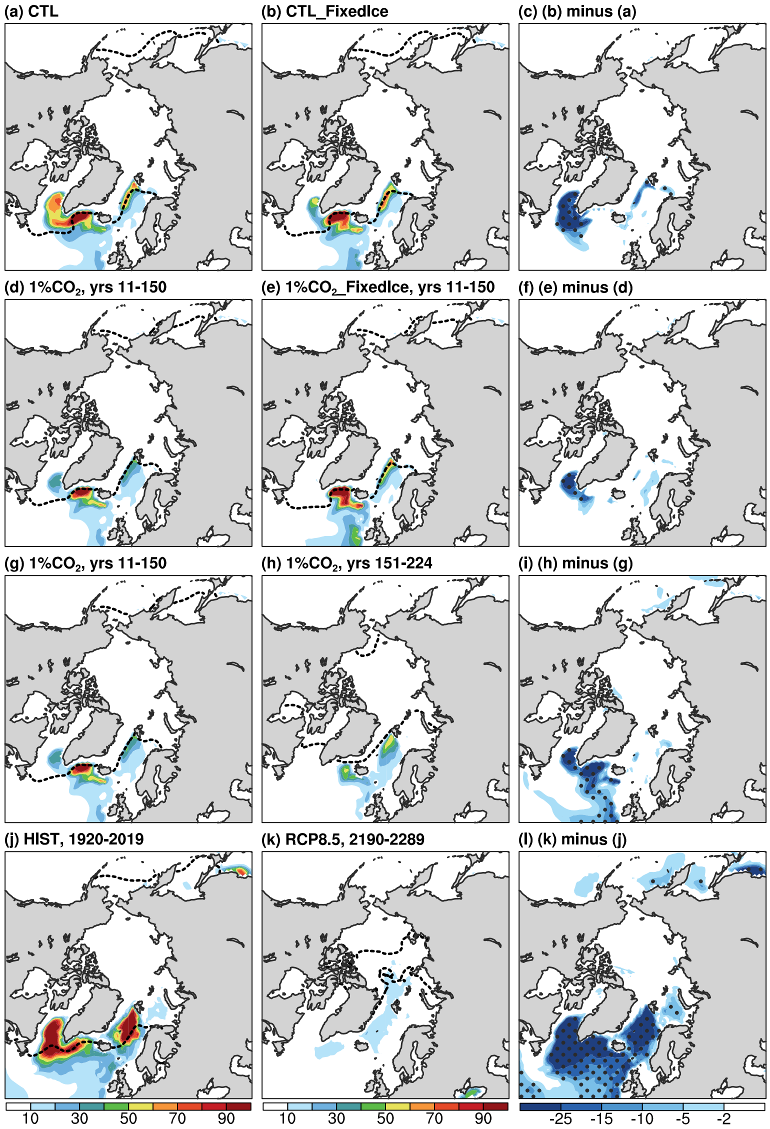

Supplementary Figure 11. Differences in multidecadal variability of ocean mixed layer depth in CESM1 and CMIP5/6 simulations. (**a**–**c**) Distributions of the standard deviation (SD) of the 10–90-year band-pass filtered DJF-mean anomalies of mixed layer depth (MLD; in m) north of 50°N from the CESM1 (**a**) CTL and (**b**) CTL_FixedIce runs and (**c**) their difference (**b** minus **a**) from years 11–490. (**d**–**f**) Same as (**a**–**c**), but for the MLD SD from the CESM1 (**d**) 1%CO_2 and (**e**) 1%CO_2_FixedIce runs and (**f**) their difference (**e** minus **d**) during years 11–150 with the forced signal removed. (**g**–**i**) Same as (**a**–**c**), but for the MLD SD from the CESM1 1%CO_2 run during (**g**) years 11–150 and (**h**) years 151–224 with the forced signal removed and (**i**) their difference (**h** minus **g**). (**j**–**l**) Same as (**g**–**i**), but for the MLD SD averaged over five CMIP5 and CMIP6 models during (**j**) 1920–2019 and (**k**) 2190–2289 (see Supplementary Table 1 for data availability) with the forced signal removed and (**l**) their difference (**k** minus **j**). The black dashed contour in the left and middle columns denotes the corresponding climatological DJF-mean sea-ice edge (for sea-ice concentration=10%) over the respective time period. The stippling in the right column indicates that the SD difference is statistically significant at the 5% level based on a *F*-test in panels (**c**,**f**,**i**) and that at least 4 out of 5 models agree on the sign of change in panel (**l**).

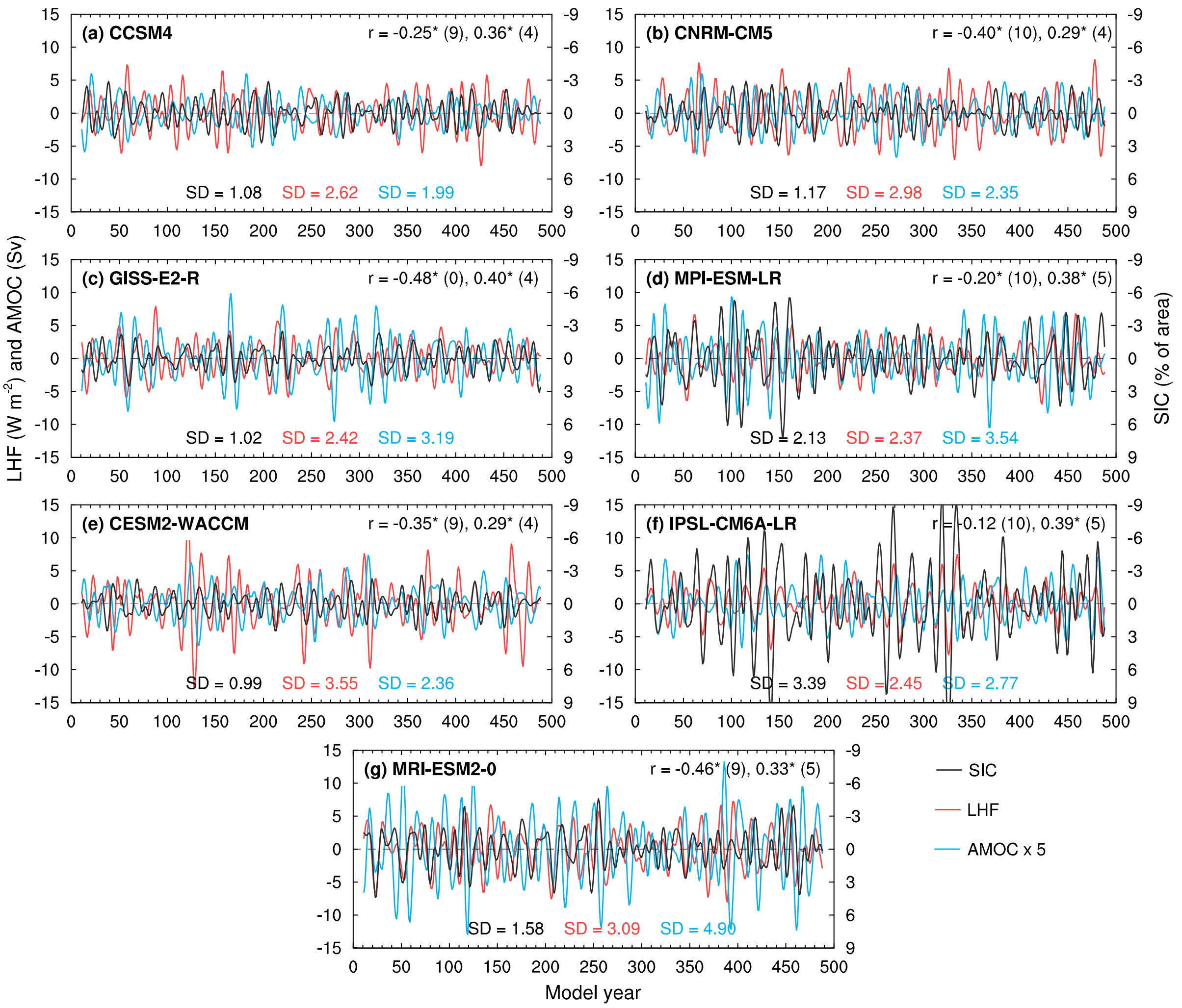

Supplementary Figure 12. Multidecadal fluctuations in regional sea-ice cover (SIC), latent heat flux (LHF), and AMOC in CMIP5/6 piControl simulations. Filtered time series of the DJF-mean anomalies in SIC (black, in % of area; right *y*-axis, increases downward) and LHF (positive upward; red, in W m^{-2}; left *y*-axis) averaged over the Labrador Sea and Davis Strait (LSDS, defined in Fig. 1b), and the AMOC index (the same definition as in Fig. 5; blue line, in Sv; multiplied by 5 to use the same left *y*-axis) during years 11–490 based on one 500-year pre-industrial control (piControl) simulation from seven CMIP5 and CMIP6 models: (**a**) CCSM4, (**b**) CNRM-CM5, (**c**) GISS-E2-R, (**d**) MPI-ESM-LR, (**e**) CESM2-WACCM, (**f**) IPSL-CM6A-LR, and (**g**) MRI-ESM2-0. A 10–90-year Lanczos band-pass filter was used. The peak correlation coefficients (*r*) are, from left to right, between SIC and AMOC and between SHF and AMOC at the lag in the parentheses during years 11–490. The SD of each curve is also given in the corresponding color for each panel. The superscript "*" ("#") indicate the correlation is statistically significant at the 5% (10%) level based on a resampling technique (see Methods).

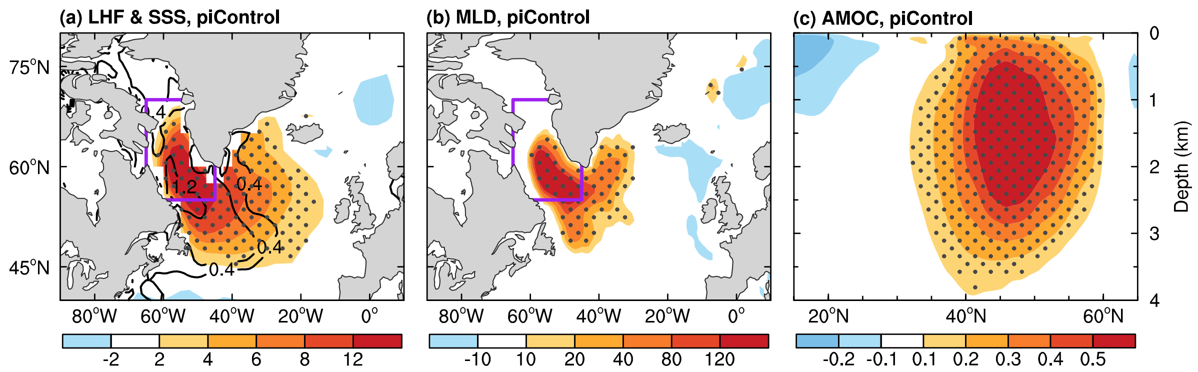

Supplementary Figure 13. Composite differences associated with multidecadal latent heat flux (LHF) anomalies in CMIP5/6 piControl simulations. Composite differences of the 10–90-year band-pass filtered DJF-mean anomalies of (**a**) LHF (positive upward; shading, in W m^{-2}) and sea surface salinity (SSS, contours, in 0.1 psu), (**b**) ocean mixed layer depth (MLD, in m), and (**c**) the 3–5-year lagged zonal-mean AMOC stream-function (shading, in Sv) between years with high (local maximum greater than +1 SD) and low (local minimum smaller than –1 SD) LHF (i.e., high LHF years minus low LHF years) over the Labrador Sea and Davis Strait (outlined in **a**, **b** as in Fig. 1b) averaged over seven 500-year piControl simulations from seven CMIP5 and CMIP6 models (except five models for MLD in **b**; see Supplementary Table 1 for data availability) during years 11 to 490. A nine-point spatial smoothing was also applied in all panels. The stippling indicates that at least 5 out of 7 (3 out of 5 for MLD in **b**) models agree on the sign of change.

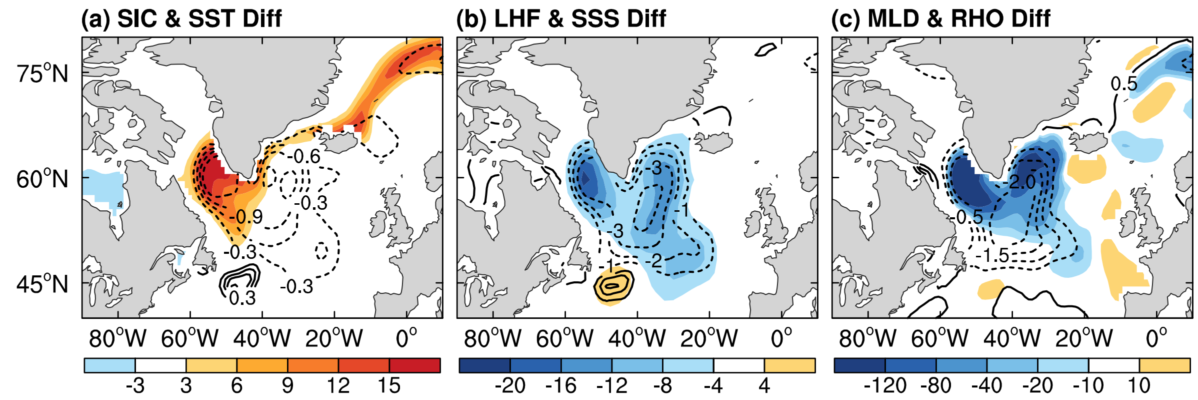

Supplementary Figure 14. CESM1-simulated climatological DJF-mean differences in sea-ice cover (SIC) and other oceanic fields over the North Atlantic region. The CTL_FixedIce-minus-CTL difference in the climatological DJF-mean (**a**) SIC (shading, in % of area) and SST (contours, in °C), (**b**) latent heat flux (LHF, shading, positive upward, in W m^{-2}) and sea surface salinity (SSS, contours, in 0.1 psu), and (**c**) ocean mixed layer depth (MLD, shading, in m) and surface ocean density (RHO, contours, in 0.1 kg m^{-3}) during years 1–500.

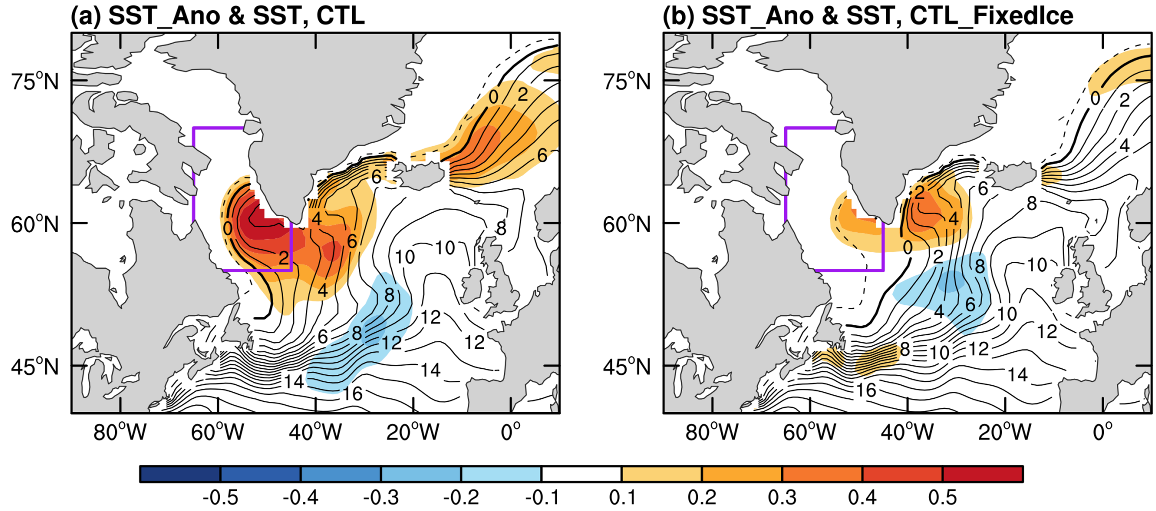

Supplementary Figure 15. Composite mean and anomalous SST during years with large latent heat flux (LHF) anomalies in CESM1. The DJF-mean SST (contours, in ºC) and 10–90-year band-pass filtered SST anomalies (shading, in ºC) averaged over the years with high LHF (local maximum higher than +1 SD) over the LSDS region (outlined in **a** and **b**) based on the CESM1 (**a**) CTL and (**b**) CTL_FixedIce runs from years 11 to 490. A nine-point spatial smoothing was applied in all panels.

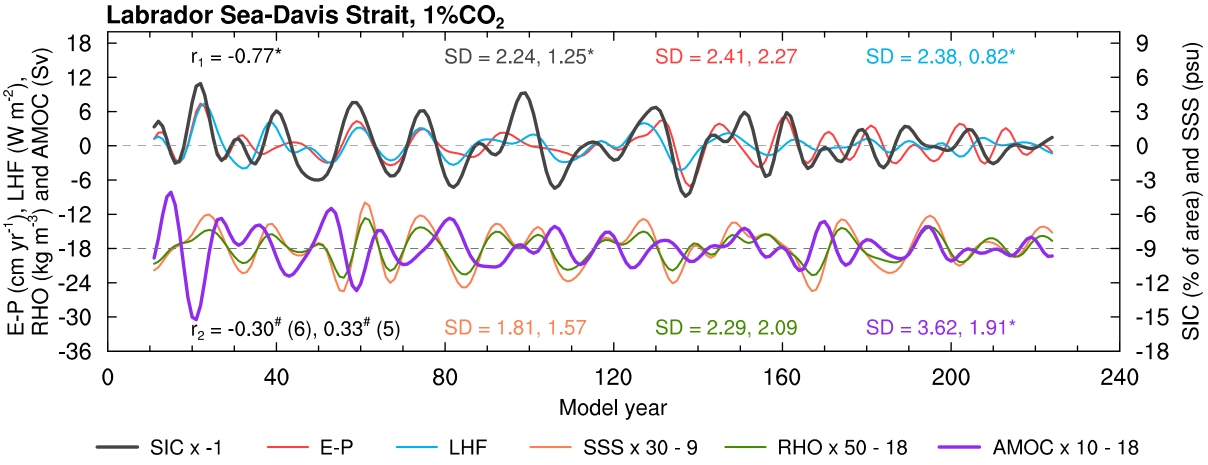

Supplementary Figure 16. CESM1-simulated multidecadal fluctuations in regional sea-ice cover (SIC), AMOC and other oceanic variables under increasing CO_2. Filtered time series of the DJF-mean anomalies (with the forced signal removed) in SIC (black, in % of area, sign reversed; right *y*-axis), evaporation minus precipitation (E–P, red, in cm year^{-1}, left *y*-axis), latent heat flux (LHF, positive upward; blue, in W m^{-2}, left *y*-axis), sea surface salinity (SSS, orange, in psu, multiplied by 30 and shifted downward by 9 to use the same right *y*-axis), and surface ocean density (RHO, green, in kg m^{-3}, multiplied by 50 and shifted downward by 18 to use the same left *y*-axis) averaged over the LSDS (defined in Fig. 1b), and the AMOC index (the same definition as in Fig. 5; purple, in Sv, multiplied by 10 and shifted downward by 18 to use the same left *y*-axis) from the CESM1 1%CO_2 run during years 11–224. A 10–90-year Lanczos band-pass filter was used. The correlation coefficient (r_1) is between SIC and LHF, and r_2 are the peak correlation coefficients, from left to right, between SIC and AMOC and between SHF and AMOC at the lag in the parentheses. The SD of each curve is given in the corresponding color, from left to right, is for years 11–150 and years 151–224. The superscript "*" and "#" indicate the correlation (the SD difference) is statistically significant at the 5% and 10% levels, respectively, based on a resampling technique (a *F*-test) (see Methods).

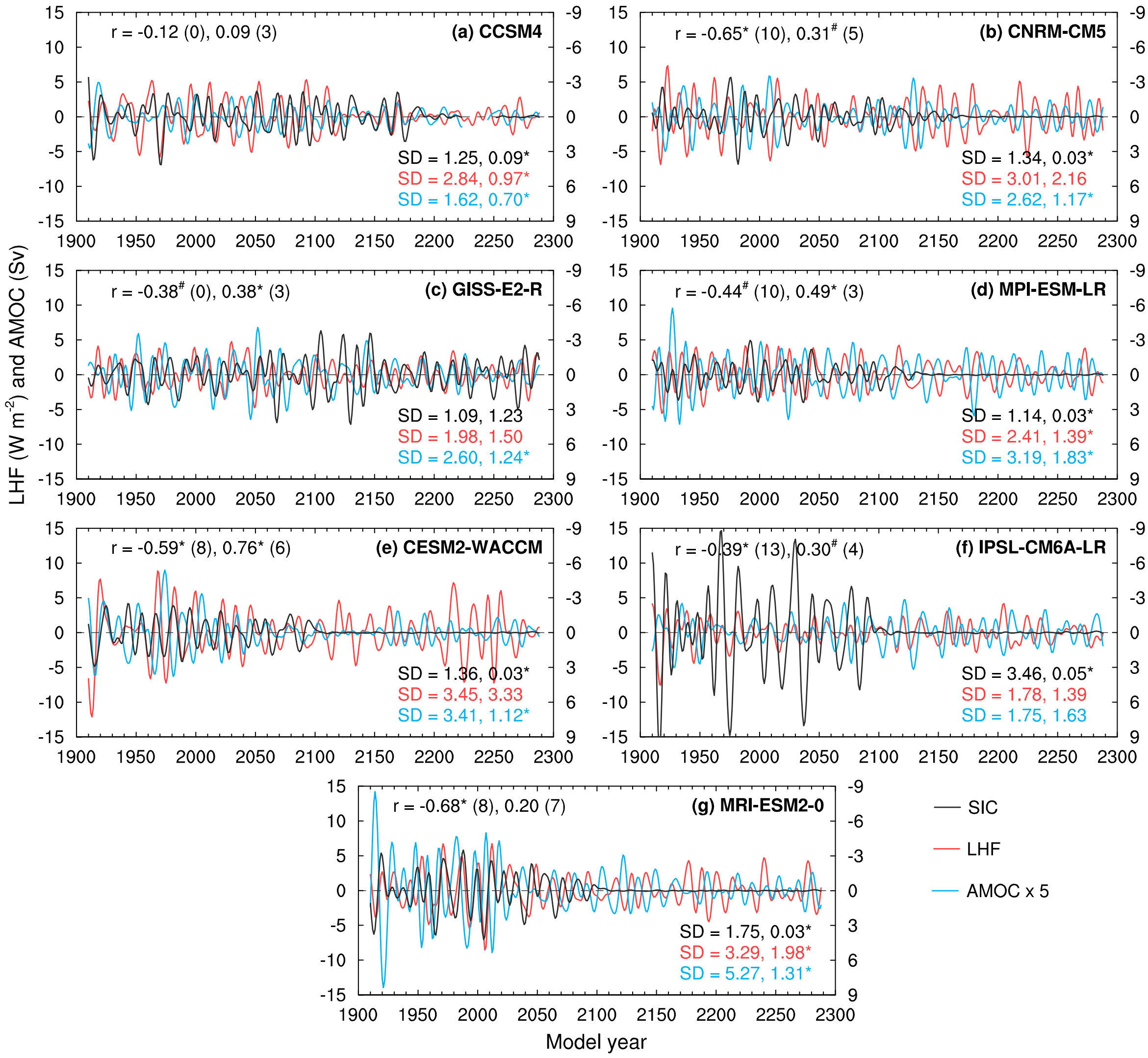

Supplementary Figure 17. Multidecadal fluctuations in regional sea-ice cover (SIC), latent heat flux (LHF), and AMOC in CMIP5/6 historical and extended RCP8.5 simulations. Filtered time series of the DJF-mean anomalies (with the forced signal removed) in SIC (black, in % of area; right *y*-axis, increases downward) and LHF (positive upward; red, in W m^{-2}; left *y*-axis) averaged over the LSDS (defined in Fig. 1b), and the AMOC index (the same definition as in Fig. 5; blue, in Sv; multiplied by 5 to use the same left *y*-axis) during 1910–2289 based on one simulation from seven CMIP5/CMIP6 models: (**a**) CCSM4, (**b**) CNRM-CM5, (**c**) GISS-E2-R, (**d**) MPI-ESM-LR, (**e**) CESM2-WACCM, (**f**) IPSL-CM6A-LR, and (**g**) MRI-ESM2-0. A 10–90-year Lanczos band-pass filter was used. The peak correlation coefficients (*r*) are, from left to right, between SIC and AMOC and between LHF and AMOC at the lag shown in the parentheses during 1920–2019; and the SIC-AMOC correlations degrade after ~2100 due to low SIC. The SDs of each curve during 1920–2019 and 2190–2289 are also given from left to right at the lower-right corner of each panel with the respective color. The superscript "*" and "#" indicate the correlation (the SD difference) is statistically significant at the 5% and 10% levels, respectively, based on a resampling technique (a *F*-test) (see Methods).

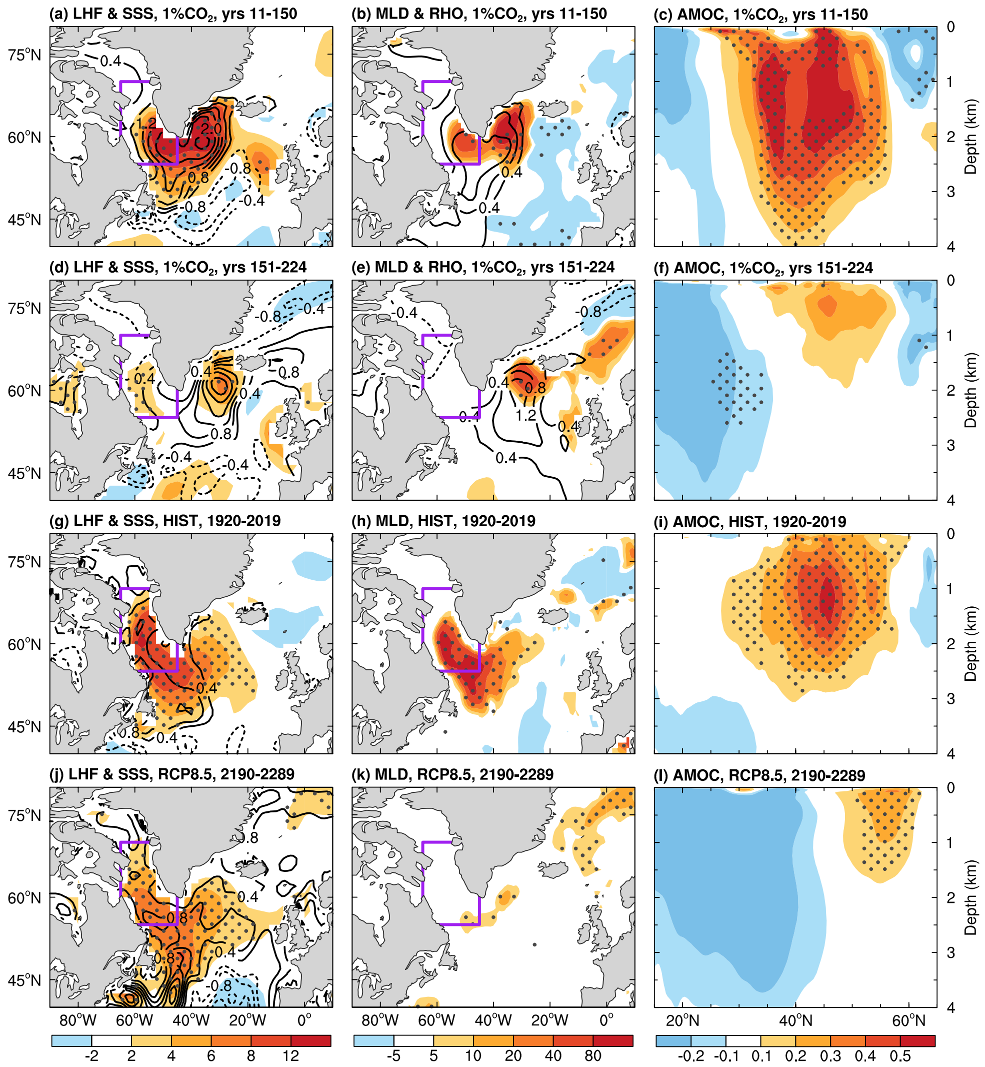

Supplementary Figure 18. Composite differences associated with multidecadal latent heat flux (LHF) anomalies under warm climates in CESM1 and CMIP5/6 simulations. (a–f) Composite differences of the 10–90-year band-pass filtered DJF-mean anomalies of (**a**, **d**) LHF (positive upward; shading, in W m^{-2}) and sea surface salinity (SSS, contours, in 0.1 psu), (**b**, **e**) ocean mixed layer depth (MLD, shading, in m) and surface ocean density (RHO, contours, in 0.1 kg m^{-3}), and (**c**, **f**) the 3–5-year lagged zonal-mean AMOC stream-function (shading, in Sv) between years with high (local maximum greater than +0.5 SD) and low (local minimum smaller than –0.5 SD) LHF (i.e., high LHF years minus low LHF years) over the LSDS (outlined in **a**, **b**, **d**, **e** as in Fig. 1b) based on the CESM1 1%CO_2 run from (**a**–**c**) years 11 to 150 and (**d**–**f**) years 151 to 224 with the forced signal removed (see Methods). (**g**–**l**) Same as (**a**–**f**), but averaged over seven CMIP5 and CMIP6 models (except five models for MLD and no models for RHO; see Supplementary Table 1 for data availability) during (**g**–**i**) 1920–2019 and (**j**–**l**) 2190–2289 with the forced signal removed (see Methods). Note that the ±0.5SD range was used here for reasonable sample sizes. A nine-point spatial smoothing was also applied in all panels. The stippling indicates that the difference is statistically significant at the 5% level based on a Student's t-test in panels **a**–**f** or at least 5 out of 7 (3 out of 5 for MLD) models agree on the sign of change in panels **g**–**l**.

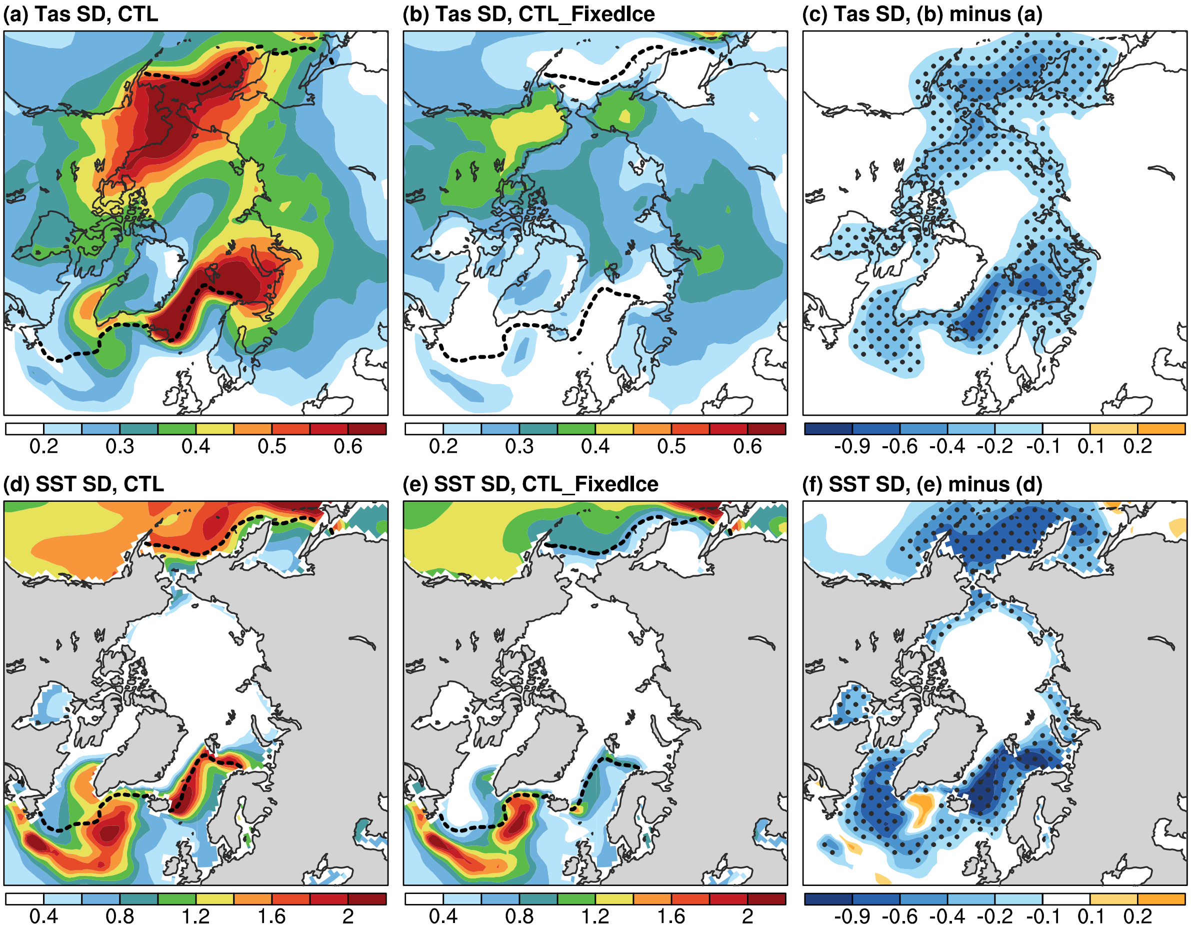

Supplementary Figure 19. CESM1-simulated multidecadal annual-mean temperature variability and its sea ice-induced difference. (**a**–**c**) Distributions of the standard deviation (SD) of the 10–90-year band-pass filtered annual-mean surface air temperature (Tas) anomalies (in °C) north of 50°N from the CESM1 (**a**) CTL and (**b**) CTL_FixedIce runs and (**c**) their difference (i.e., CTL_FixedIce minus CTL) during years 11 to 490. (**d**–**f**) Same as (**a**–**c**) but for the similarly-filtered annual-mean SST anomalies (in °C; multiplied by 5 as in Fig. 1). The black dashed contour in **a** and **d** (**b** and **e**) denotes the climatological annual-mean sea-ice edge (for sea-ice concentration=10%) over the same period from CTL (CTL_FixedIce). The stippling in **c** and **f** indicates that the SD difference is statistically significant at the 5% level based on a *F*-test. Note that results are similar to DJF except for smaller magnitudes.
